# Enhanced Synaptic Connectivity in the Dentate Gyrus during Epileptiform Activity: Network Simulation

**DOI:** 10.1155/2013/949816

**Published:** 2013-02-04

**Authors:** Keite Lira de Almeida França, Antônio-Carlos Guimarães de Almeida, Antonio Fernando Catelli Infantosi, Mario Antônio Duarte, Gilcélio Amaral da Silveira, Fulvio Alexandre Scorza, Ricardo Mario Arida, Esper Abrão Cavalheiro, Antônio Márcio Rodrigues

**Affiliations:** ^1^Laboratório de Neurociência Experimental e Computacional, Departamento de Engenharia de Biossistemas, Universidade Federal de São João del-Rei (UFSJ), Brazil; ^2^Programa de Engenharia Biomédica, Universidade Federal do Rio de Janeiro (UFRJ/COPPE), Brazil; ^3^Disciplina de Neurologia Experimental, Escola Paulista de Medicina (EPM), Universidade Federal de São Paulo (UNIFESP), Brazil; ^4^Disciplina de Neurofisiologia e Fisiologia do Exercício, Escola Paulista de Medicina (EPM), Universidade Federal de São Paulo (UNIFESP), Brazil

## Abstract

Structural rearrangement of the dentate gyrus has been described as the underlying cause of many types of epilepsies, particularly temporal lobe epilepsy. It is said to occur when aberrant connections are established in the damaged hippocampus, as described in human epilepsy and experimental models. Computer modelling of the dentate gyrus circuitry and the corresponding structural changes has been used to understand how abnormal mossy fibre sprouting can subserve seizure generation observed in experimental models when epileptogenesis is induced by status epilepticus. The model follows the McCulloch-Pitts formalism including the representation of the nonsynaptic mechanisms. The neuronal network comprised granule cells, mossy cells, and interneurons. The compensation theory and the Hebbian and anti-Hebbian rules were used to describe the structural rearrangement including the effects of the nonsynaptic mechanisms on the neuronal activity. The simulations were based on neuroanatomic data and on the connectivity pattern between the cells represented. The results suggest that there is a joint action of the compensation theory and Hebbian rules during the inflammatory process that accompanies the status epilepticus. The structural rearrangement simulated for the dentate gyrus circuitry promotes speculation about the formation of the abnormal mossy fiber sprouting and its role in epileptic seizures.

## 1. Introduction

Epileptic syndromes are a group of neurological disorders with different etiology and clinics characterized by recurrent seizures with excessive and hypersynchronous neuronal activity [1]. Nowadays, approximately 50 million people are affected with epilepsy and 40% of which are classified as temporal lobe epilepsy (TLE) [2].

The human TLE is frequently associated with neuronal loss in the hippocampus and dentate gyrus (DG) [3]. Throughout this degeneration, called hippocampal sclerosis, the granule cells are less affected. The neurotransmitter gamma-aminobutyric acid and proteins expressed in these cells are allegedly responsible for neuroprotection, increasing resistance to excitatory injury [4]. The DG has been proposed to be a gate for entry of intense neuronal activity into the hippocampus. However, in TLE, the synaptic reorganization involving the DG granule cells (abnormal sprouting of hippocampal mossy fibers) may act to reduce the DG filtration properties, increasing the propensity to intensify the neuronal activity and, therefore, the seizure propagation through the hippocampus and other limbic system structures [5].

Mossy fiber sprouting (MFS) has been observed in several animal models of TLE. The recurrent synaptic transmission between granule cells is one of the supposed inductors of a recurrent excitation between these cells and therefore is responsible for the generation of spontaneous seizures with probable origin in the hippocampus [8, 9]. Some investigators believe that the MFS makes the hippocampal circuitry susceptible to prolonged bursts of action potentials, contributing to the seizure onset [9, 10]. Thus, the abnormal MFS could play an important role in sustaining seizures in TLE with mesial sclerosis, contributing to the provision of the epileptogenic substrate of the hippocampus. According to Lytton et al. [11], the abnormal organization of the brain circuits can be considered crucial for several types of epilepsy. 

On the other hand, the occurrence or lack of occurrence of spontaneous recurrent seizures in an animal model is not directly related to the presence of mossy fiber sprouting. Therefore, the complexity involved in the investigation of the main mechanisms responsible for seizure eruption makes the computational simulation of DG an indispensable approach in providing new insights. Of course any model is an oversimplification of the reality. In the particular case of a mathematical model, the reductionism must be a rule to make the computational implementation reliable. However, it is a powerful tool to help evaluate if the minimum conditions represented are enough to reproduce the phenomenon investigated.

In 1943, McCulloch and Pitts created the first formalized model of an artificial neural network. Since then, computer modeling has enabled important advances in the understanding of complex phenomena such as epilepsy [12–14].

The aim of this study was to investigate the possible role of abnormal MFS on the DG excitability, involved in experimental models of *status epilepticus*. The computational model was based on the McCulloch-Pitts formalism and neuroanatomical data as well as on patterns of connectivity of the main cell types present in the hippocampus [12, 15–19]. The model also represents sustained depolarizations that are typical of nonsynaptic mechanisms during the ictal periods and changes in GABAa transmission reported during inflammatory response observed after pilocarpine-induced seizures. The simulations create the possibility for discussion concerning the complex role of the synaptic reorganization associated with experimental models of *status epilepticus*.

## 2. Methods

### 2.1. Neuronal Network Model

**Figure 1:**
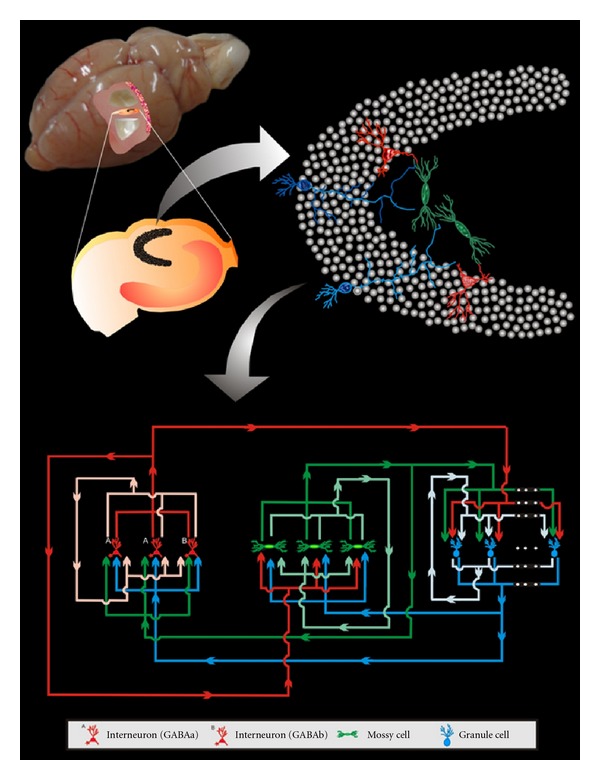
Diagrammatic representation of the basic circuitry of the dentate gyrus represented in the model.

 The neural network implemented for the simulation study is based on the McCulloch-Pitts formalism [15, 20], including the Compensation Algorithm for synaptogenesis, proposed by Dammasch et al. [19, 21], and Hebbian and anti-Hebbian rules. The cell population represents the main types described in the dentate gyrus (DG) as well as its connectivity (Figure 1). Since the cytoarchitecture of DG is favourable to the non-synaptic connections, they were also represented in the model.

 The neuronal network, with *N* neurons, is composed of NE excitatory and *N* − NE inhibitory neurons. The excitatory neurons were divided into {1,…, NE − 3} granule cells and {NE + 2,…, NE} hilar mossy cells. The connectivity is represented by a connection matrix **C**(*N* × *N*), where *c*_*i*,*j*_, 1 ≤ *i*, *j* ≤ *N* is the synaptic weight of the connection of the neuron *j* on the neuron *i*.

The collection of states of each neuron of the network is represented by a vector *z* ^*t*^ = (*z*_1_^*t*^,…, *z*_*N*_^*t*^), where *z*_*i*_^*t*^ ∈ {0,1}, 1 ≤ *i* ≤ *N*, *t* = 0, 1, 2,…. Each neuron *i* may have two possible states in the instant of time *t*: active (*z*_*i*_^*t*^ = 1) or inactive (*z*_*i*_^*t*^ = 0). The probability of neuron *i* turning active in time *t* could be estimated by the threshold function [20]:

(1) $$\begin{array}{c} \text{prob}\left(z_{i}^{t}\right)=\frac{1}{1+e^{\left(\text{M}{\text{P}}_{i}^{t-1}+\alpha -\theta \right)/-\beta }}, \end{array}$$

where *α* represents the stimulation input, MP_*i*_ is the membrane potential of the neuron *i*, *θ* is the threshold, and *β* represents the noise of the threshold function. 

The membrane potential in the instant of time *t* of the neuron *i*, MP_*i*_^*t*^, is calculated by

(2) MPit=τ∑j=1NE−3ci,jzjt−1+κu∑j=NE−2NEci,jzjt−1−φ∑j=NE+1Nci,jzjt−1+NSit,

where NS_*i*_^*t*^ is the effect of the non-synaptic mechanisms on the transmembrane potential. The connection weights are *τ* (connections from granule cells), *κ*_*u*_ (*u* = 1  se  *i* ≤ NE and *u* = 2  se  *i* > NE; connections from mossy cells), and *φ* (inhibition from interneurons).

According to Almeida et al. [14, 22], the Na^+^ influx trough ionic channels and the Na^+^ efflux trough Na/K pump define the transition points between the ictal and interictal periods during non-synaptic epileptiform activity. In the present model, the ictal period of the epileptiform activities was associated with the spontaneous increase of cell activities (SICA). Representating these two Na^+^ currents in the generation of a SICA, NS_*i*_^*t*^ was calculated based on two components: NSE, representing the excitatory effect of the Na^+^ influx, and NSI, representing the inhibitory effect of the Na^+^ efflux. Therefore,

(3) $$\begin{array}{c} {\;\text{NS}}_{i}^{t}=a\cdot {\text{NSE}}_{i}^{t}-b\cdot {\text{NSI}}_{i}^{t}, \end{array}$$

where “*a*” and “*b*” are parameters used to adjust the balance between excitation and inhibition, respectively.

In the proposed model, to simulate the ictal and interictal periods, two states of the neuronal activity were identified: *sustained depolarization *state and *polarized* state. The former state comprises the ictal period and the last the interictal period. In order to represent the transition between both states, the *sustained depolarization* state was divided in two: the *sustained depolarization* itself and the *in repolarization* state. Defining *s*_*i*_^*t*^ as the average activity of the cell *i*, as described in (9), and *L* as the threshold for the action of the non-synaptic mechanisms, the *sustained depolarization* happens when *s*_*i*_^*t*^ > *L*. During the *sustained depolarization*, the neuron is firing and the sustained depolarization is done due to Na^+^ influx trough ionic channels greater than its efflux trough pump, which can be represented by NSE_*i*_^*t*^ > NSI_*i*_^*t*^. Consequently, when in the *in repolarization *state NSE_*i*_^*t*^ < NSI_*i*_^*t*^ is required, the essential condition for the interictal period (*polarized *state) is *s*_*i*_^*t*^ < *L*. The mathematical description of the three states is basically defined by the same functional composition. The changing rates of NSE and NSI follow the equations

(4) dNSEitdt=h(t)−c·NSEit,dNSIitdt=k(t)−d·NSIit,

where *h*(*t*) and *k*(*t*) are dependent on the state and *c* and *d* are time constants governing the recovering rate of NSE and NSI. To Summarize, consider the following

(i) *Sustained depolarization* state (*s*_*i*_^*t*^ > *L* and NSE_*i*_^*t*^ > NSI_*i*_^*t*^):
  (5) $$\begin{array}{c} h\left(t\right)=e\cdot \left(s_{i}^{t}-0.25\right),\\ k\left(t\right)=f\cdot {\text{NSE}}_{i}^{t} \end{array}$$
  with *f* constant and *e* dependent on NSE_*i*_^*t*^, according to
  (6) $$\begin{array}{c} e=\overset{-}{e}\cdot \left(s_{i}^{t}-0.25\right)\cdot \left(1-\tanh\left(\frac{{\text{NSE}}_{i}^{t}-18}{0.5}\right)\right), \end{array}$$
  where $\overset{-}{e}$ is constant. In this state, *h*(*t*) represents an increment of the Na^+^ influx through the channels, based on the increment of the neuronal discharge. On the other side, the increment of intracellular Na^+^ increases the Na/K pump action, promoting the inhibitory effect of the electrogenic current, which is represented in the present model by an effect proportional to NSE.
(ii) *In repolarization *state (NSE_*i*_^*t*^ < NSI_*i*_^*t*^):
  (7) $$\begin{array}{c} h\left(t\right)=-g\cdot R_{\text{NSE}}^{t},\;\;\;\text{if}\;{\text{NSE}}_{i}^{t}>0.1,\\ k\left(t\right)=-h\cdot R_{\text{NSI}}^{t},\;\;\;\text{if}\;{\text{NSI}}_{i}^{t}>0.009, \end{array}$$
  where *g* and *h* are constants and *R*_NSE_^*t*^ and *R*_NSI_^*t*^ are functions that contribute to cell repolarization:
  (8) $$\begin{array}{c} \frac{{dR}_{\text{NSE}}^{t}}{dt}=l\cdot \left(1-R_{\text{NSE}}^{t}\right),\;\text{if}\;{\text{NSE}}_{i}^{t}>0.1,\\ R_{\text{NSE}}^{t}=0,\;\text{if}\;{\text{NSE}}_{i}^{t}\leq 0.1,\\ \frac{{dR}_{\text{NSI}}^{t}}{dt}=m\cdot \left(1-R_{\text{NSI}}^{t}\right),\;\text{if}\;{\text{NSI}}_{i}^{t}>0.009,\\ R_{\text{NSI}}^{t}=0,\;\text{if}\;{\text{NSI}}_{i}^{t}\leq 0.009, \end{array}$$
  where *l* and *m* are constants. The aim of the above equations was to represent the moment when the electrogenic current of the Na/K pump is able to interfere with the membrane potential promoting the repolarization. In these circumstances, the voltage-dependent sodium channels start to reduce conductance and the repolarization takes place. Equation (9) is a phenomenological representation of this behavior.
(iii) *Polarized *state (*s*_*i*_^*t*^ > *L*) with *h*(*t*) = *k*(*t*) = 0.

### 2.2. Neuronal Network Connectivity

#### 2.2.1. Compensation Theory

Experimental observations indicate that neurons react to anatomical changes induced by long-term metabolic disequilibrium. These reactions depend on whether the state is high or low [19, 20, 23]. Therefore, each neuron follows the local rules aiming at compensatory changes to the input activity, caused by structural adaptations. Consequently, for a specific neuron *i*, its deviation from the average activity expected, in the present work 0.2, is called the morphogenetic state of the neuron, Δ*s*_*i*_^*t*^, which is estimated by means of the following relationship:

(9) $$\begin{array}{c} \Delta s_{i}^{t}=s_{i}^{t}-0.2,\;\text{where}\;s_{i}^{t}=\frac{\sum_{t}^{t+\Delta t}\text{prob}\left(z_{i}^{t}\right)}{\Delta t}, \end{array}$$

where the average activity is calculated from *t* to *t* + Δ*t* and Δ*t* is the morphogenetic time step, corresponding to the period of time needed to perform the connectivity changes of the network. *s*_*i*_^*t*^, used as a measure of the neuron *i* activity, is the average firing probability.

The morphogenetic state of neuron represents the neuronal capacity to form, stabilize, or degrade pre- or postsynaptic elements or synapses [20, 21]. The possible interactions between these three states follow the synaptogenesis compensation theory proposed by Wolff and Wagner [17]. According to this theory, the afferent synapse spectrum influences—via transmission—the functional state of the neuron, estimated as an average of activity along the determinate period of time Δ*t*. Depending on its level of activity, the functional state leads to a morphogenetic state that tries to compensate its deviations. This is performed by changing the neuronal elements, bound and free, pre- and postsynaptic, after each morphogenetic time step. The degeneration of bound elements and recombination of free synaptic elements lead to a new structural state. The new afferent spectrum leads the neuron to a new functional state.

To simulate the network morphogenesis, the synapses are distinguished in pre- (pr) and postsynapses (po), which are then divided into bound (b) and free (f). The postsynaptic elements have additional divisions: excitatory (e) and inhibitory (i) [20]. This classification leads to the following representation:

- bound (bpr) and free (fpr) presynaptic elements;
- bound (bepo) and free (fepo) excitatory postsynaptic elements;
- bound (bipo) and free (fipo) inhibitory postsynaptic elements.

The sum of the synaptic elements of the neuron *i*, according to this classification, represented by *σ*_*i*_, is defined as follows [19, 20]:

- sum of the bound excitatory postsynaptic elements: *σ*_*i*_^bepo^ = ∑_*j*=1_^NE^ *c*_*i*,*j*_,
- sum of the bound inhibitory postsynaptic elements: *σ*_*i*_^bipo^ = ∑_*j*=NE+1_^*N*^ *c*_*i*,*j*_,
- sum of the free excitatory postsynaptic elements: *σ*_*i*_^fepo^,
- sum of the free inhibitory postsynaptic elements: *σ*_*i*_^fipo^,
- sum of the bound presynaptic elements: *σ*_*i*_^bpr^ = ∑_*i*=1_^*N*^ *c*_*i*,*j*_,
- sum of free presynaptic elements: *σ*_*i*_^fpr^.

The morphogenetic rules depend on the morphogenetic state of the neuron Δ*s*_*i*_, on the actual structural state of the neuron, and also on the cell sensitivity to structural variations [19, 20]. These rules are represented in Table 1. 

The current structural state of the neuron is characterized by the number of afferent and efferent synapses. The synaptogenesis and the modification of the synaptic contact can change the structural state of the network [19]. According to the formalization of the compensation theory of synaptogenesis [17], two kinetic parameters are defined: *k*_⋯_^*H*^ and *k*_⋯_^*L*^, where *H* and *L* refer to the level of excitability of the neuron: high (*H*) and low (*L*). Changes in the amount of synaptic elements of the neuron *i*, Δ*σ*_*i*_, are calculated based on the dependence of the level of excitability (*k* ^*H*^ and *k* ^*L*^ are proportionality constants), on the neuron's deviation from a desired medium average activity (Δ*s*_*i*_^*t*^) and on the current amount of synaptic elements (Table 1). In the present model, the changes in the connectivity of the network followed the rules proposed by Dammasch et al. [19] and Dammasch et al. [21].

The network connectivity variation also happens with dependence on the reduction of the bound synaptic elements. The presynaptic elements' decay is proportional to the strength of the actual connections [19]:

(10) $$\begin{array}{c} c_{i,j}=c_{i,j}+\left(\Delta \sigma_{j}^{\text{bpr}}\frac{c_{i,j}}{\sigma_{j}^{\text{bpr}}}\right),\;\forall c_{i,j}\in C. \end{array}$$

The loss of postsynaptic elements is computed separately for inhibitory and excitatory synapses, according to the following equation [20]:

(11) $$\begin{array}{c} c_{i,j}=c_{i,j}+\left\{\begin{array}{c} \Delta \sigma_{j}^{\text{bepo}}\frac{c_{i,j}}{\sigma_{j}^{\text{bepo}}},\;j=1,\ldots ,\text{NE}\\ \Delta \sigma_{j}^{\text{bipo}}\frac{c_{i,j}}{\sigma_{j}^{\text{bipo}}},\;j=\text{NE}+1,\ldots ,N \end{array}\right\}, \end{array}$$

for all *c*_*i*,*j*_ ∈ **C**.

The presynaptic elements of altered synapses are redistributed postsynaptically while the postsynaptic elements are removed. Therefore, the amount of presynaptic elements previously bound (equal to the amount of degraded postsynaptic elements |*δ*_*i*,*j*_^post^|) is transferred to the amount of free presynaptic elements [20]:

(12) $$\begin{array}{c} \sigma_{j}^{\text{fpr}}=\sigma_{j}^{\text{fpr}}+{\Delta \sigma }_{j}^{\text{fpr}}+\sum_{i=1}^{N}\left|\delta_{i,j}^{\text{post}}\right|,\\ \sigma_{i}^{\text{fepo}}=\sigma_{i}^{\text{fepo}}+{\Delta \sigma }_{i}^{\text{fepo}},\\ \sigma_{i}^{\text{fipo}}=\sigma_{i}^{\text{fipo}}+{\Delta \sigma }_{i}^{\text{fipo}}. \end{array}$$

New synaptic contacts and the reinforcement of the current synaptic contacts take place from the recombination of free elements. The recombination is given by Butz et al. [20]:

(13) ci,j=ci,j+{σjfprσifepomax⁡(∑i=1Nσifepo,∑i=1NEσifpr), j=1,…,NEσjfprσifipomax⁡(∑i=1Nσifipo,∑j=NE+1Nσjfpr), j=NE+1,…,N}≥0,

for all *c*_*i*,*j*_ ∈ **C**, where *i* = 1,…, *N*.

The amount of free synaptic elements remained during the recombination is also renewed subsequently. 

#### 2.2.2. Hebbian and Anti-Hebbian Rules

The synaptic modification during learning is made by a mechanism that depends on the simultaneous activity of the presynaptic button and the postsynaptic cell. According to the Hebbian learning rule [24], a synapse is potentiated when the activity in the synaptic button increases at the same time that the postsynaptic neuron is depolarized. The synaptogenesis, according to the Hebbian rules depend on the activities of connected cells and on the contribution of each cell to the firing of the other. Therefore, the change of the connection weight between two neurons, *i* and *j*, is determined by

(14) $$\begin{array}{c} \Delta c_{ij}^{t}=\varepsilon \Delta s_{i}\Delta s_{j}\eta_{ij},\;\;\text{if}\;s_{i}^{t}>0.25,\;\;s_{j}^{t}>0.25,\\ \Delta c_{ij}^{t}=0,\;\text{otherwise}, \end{array}$$

where *ε* is constant and *η*_*ij*_ is the number of times that the discharge of the presynaptic neuron *j* is followed by discharge of the postsynaptic neuron *i* during one morphogenetic time step. Assuming that the formation of new synapses is limited as well as the strengthening of the synaptic weight, in the present model a sigmoid function was used to calculate *ε*

(15) $$\begin{array}{c} \varepsilon_{ij}^{t}=\frac{\rho }{{1+e}^{\left(c_{ij}^{t}-0.4\right)/0.05}}, \end{array}$$

where *ρ* is a proportionality constant.

When the postsynaptic terminals are active and the presynaptic neuron remains inactive, the corresponding synapses are depressed. This process is known as anti-Hebbian plasticity, because of its antagonism in respect to the Hebb learning. Thus, the changes in the connectivity were performed according to

(16) $$\begin{array}{c} \Delta c_{ij}^{t}=-\mu \Delta s_{i}\Delta s_{j}\lambda_{ij},\;\text{if}\;s_{i}^{t}>0.25,\;\;s_{j}^{t}>0.25,\\ \Delta c_{ij}^{t}=0,\;\text{otherwise}, \end{array}$$

where *μ* is constant and *λ*_*ij*_ is the number of times that the discharge of the postsynaptic neuron *i* is not preceded by discharge of the pre-synaptic neuron *j* during one morphogenetic time step.

### 2.3. Connection Matrix and Parameters of the Model

The concept of the connection matrix representing the DG was based on the current literature. According to Dyhrfjeld-Johnsen et al. [16], the hippocampus comprises 1.000.000 granule cells, 30.000 mossy cells, 10.000 basket cells, 2.000 axo-axonic cells, 4.000 MOPP cells (molecular layer perforant path-associated cell), 12.000 HIPP cells (hilar perforant path-associated cell), 3.000 HICAP cells (hilar commissural-associational pathway related cells), and 3.000 IS cells (interneuron selective cells). The model network was performed in a reduced scale (1 : 10.000), that is, with 99 granule cells, 3 mossy cells, and 3 interneurons (basket, axoaxonic, MOPP, HIPP, HICAP, and IS). Combining these three types of cells, nine groups of connections were represented and the connections' arrangement for the initial conditions of the simulations was set according to experimental investigations [12, 16]:

- group I (granule cells → granule cells): no connections;
- group II (granule cells → mossy cells): each mossy cell receives connection from 33 granule cells;
- group III (granule cells → interneurons): each interneuron receives connection from 33 granule cells;
- group IV (mossy cells → granule cells): each mossy cell sends a connection to all granule cells;
- group V (mossy cells → mossy cells): each mossy cell sends a connection to all mossy cells;
- group VI (mossy cells → interneurons): each mossy cell sends a connection to all interneurons;
- group VII (interneurons → granule cells): each interneuron sends a connection to all granule cells;
- group VIII (interneurons → mossy cells): each interneuron sends a connection to all mossy cells;
- group IX (interneurons → interneurons): each interneuron sends a connection to all interneurons.

The initial values of the elements of the connectivity matrix followed a normal distribution with mean and standard deviation set to 1.0 ± 0.2. 

According to Dammasch et al. [19], Dammasch et al. [21], and Cromme and Dammasch [23], it is hypothetically assumed that degeneration of the free synaptic elements is faster than the degeneration of the bound synaptic elements. Dammasch et al. [21] found in simulations that while the relation between decrease and increase of bound elements is critical for stability, the decrease of free elements is of less importance. Therefore, the following rations of kinetic parameters were chosen:

(17) $$\begin{array}{c} k_{\text{bpr}}^{L}=k_{\text{fpr}}^{H}\cdot 0.033,\\ k_{\text{bpr}}^{L}=k_{\text{fpr}}^{L}\cdot 0.167. \end{array}$$

To maintain the oscillation properties of the network during the morphogenesis, as proposed by Cromme and Dammasch [23], it was assumed that

(18) $$\begin{array}{c} k_{\text{fepo}}^{L}=9*k_{\text{fipo}}^{H}. \end{array}$$

Assuming no bias decrease of the synaptic elements, then

(19) $$\begin{array}{c} k_{\text{fepo}}^{H}=k_{\text{fipo}}^{L}=k_{\text{fpr}}^{L},\\ k_{\text{bepo}}^{H}=k_{\text{bipo}}^{L}=k_{\text{bpr}}^{L}. \end{array}$$

The relationships of the kinetic parameters described above allow the description of one parameter in terms of the other. Choosing *k*_fipo_^*H*^ as the adjusting parameter,

(20) $$\begin{array}{c} k_{\text{fipo}}^{H}=v, \end{array}$$

where *v* can be interpreted as a constant that defines the speed of the morphogenetic changes. During the process of network connectivity change, the kinetic parameters must guarantee smooth convergence in the change of synaptic elements [21] and thus *v* must be small. In this model, studying the effect of the compensation theory on the network activity, *v* was set in the interval 0 ≤ *v* ≤ 0.1. Taking into account the investigations by Dammasch and Wagner [18], Dammasch et al. [19], Dammasch et al. [21], Cromme and Dammasch [23], and Butz et al. [20], it was assumed that *φ* = 8, *β* = 2, and *θ* = 1.0.

For the excitatory pre-synaptic cells the synaptic weights were *τ* = 0.19, *κ* = *κ*_1_ = 4.92, when *j* < NE − 3, and *κ* = *κ*_2_ = 2.37, when NE − 3 < *j* ≤ NE. These parameters were estimated by running simulations of the network activity without stimulation and allowing it to reach stable activity [19]. The network was considered stable when the average activity reaches values in the range 0.15–0.25.

The parameters related to the increase of the neuronal activity due to the nonsynaptic mechanisms were adjusted to induce depolarization and repolarization during SICA mimicking the effects of the Na^+^ influx through the channels and its efflux through the pump, during a spontaneous nonsynaptic epileptiform activity, as described by Almeida et al. [14]. With this aim, the adjusted values were *a* = 0.8, *b* = 0.2, *c* = 2.8 × 10^−6^ min⁡^−1^, *d* = 5.6 × 10^−5^ min⁡^−1^, *f* = 2.8 × 10^−3^ min⁡^−1^, $\overset{-}{e}=1.1\;\min^{-1}$, *g* = 2.8 × 10^−2^ min⁡^−1^, *h* = 2.8 × 10^−2^ min⁡^−1^, *l* = 2.8 × 10^−2^ min⁡^−1^, *m* = 1.1 × 10^−2^ min⁡^−1^, and *L* = 0.32.

Two timescales were adopted in the simulations, one for the cellular activity changes, corresponding to the duration of each interaction *t*, and a timescale for the connectivity changes (duration of one morphogenetic time step Δ*t*). Thus, one morphogenetic time step of the proposed model (Δ*t*) corresponds to the spiking remodelling and, similarly, to the axonal ramification between different postsynaptic targets [25, 26]. In the model, each morphogenetic time step corresponds to 150 interactions of *t* and each interaction *t* to 0.36 min. 

The effect of the Hebbian rule on the connectivity changes and on the network activity was investigated to guarantee smooth connectivity changes. For this conditions, the possible range for *ρ*  (0 < *ρ* < 0.5) was determined. 

## 3. Results

**Figure 2:**
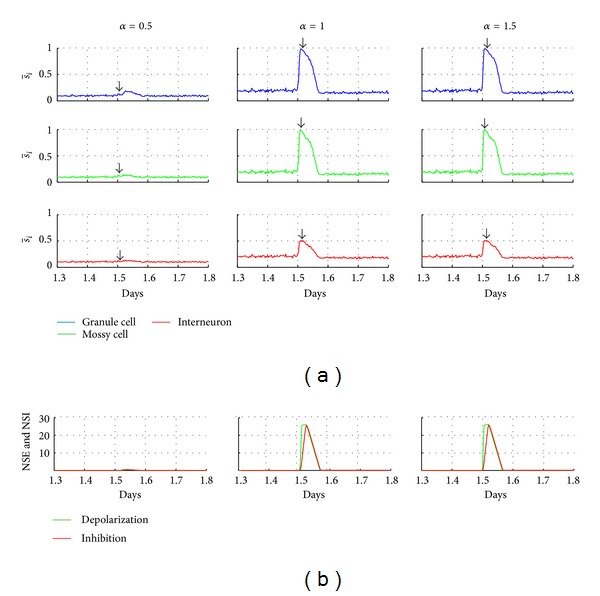
Stimulation effect on the neuronal network. (a) Average activity of the network for different types of cell in response to stimulation. (b) Curves of the nonsynaptic excitation (NSE) and inhibition (NSI) functions for the different stimulus intensities (*α* = 0.5, *α* = 1.0, and *α* = 1.5, resp., left, middle, and right).

By investigating the effect of different strengths of stimulation (*α* = 0.5, 1.0, and  1.5; duration = 70 min⁡) on the neuronal network activity, an increase of induced activity can be seen (Figure 2). It can be observed that the increase of the network connectivity also causes a progressive increase of the network activity with afterdischarges. Mimicking the nonsynaptic mechanisms, during afterdischarges, the depolarization function NSE increases quickly, also raising the *s*_*i*_ value. The inhibition function, NSI, increases more slowly and, at the end of afterdischarges, overcomes the excitation function. From that moment on, the values of NSE and NSI are restored to the levels at which the neuronal activity remains low.

To investigate how different situations of combinations of actuation of the compensation theory and the Hebbian rule act on the network, three simulations were made. The results are shown in Figure 2: (i) without stimulation; (ii) with stimulation; (iii) with stimulation and after the stimulation, assuming the transient change of the interneuron action from inhibitory to excitatory. For the stimulations performed in the simulations (ii) and (iii), the stimulus was applied at the day 1.5, with *α* = 1.0, during 70 min. The stimulus intensity was adjusted to be less intense but sufficient to induce maximal activation of the network. This procedure took into account the objective of representing the experimental protocols of drug applications in animals to induce epileptiform seizures and, consequently, the *status epilepticus* [27–29]. The transient change of the inhibitory effect of interneurons to excitatory effect aimed at representing the excitatory effect of the GABAa receptors, typical of the inflammatory states in the brain, which can persist from the first 24 h for up to two weeks after the *status epilepticus *[30]. Experimental observations show that after status epilepticus, at the beginning of the latent period, changes in the transmembrane Cl-gradients may occur, due to anionic accumulation in the neuronal cytoplasm. This accumulation is enough to change the GABAa receptors activation effect from inhibitory to excitatory. The cause of this change was demonstrated to be related to the diminished expression of the cotransporter KCC2, responsible for the Cl-extrusion [30]. This mechanism is reported by Morimoto et al. [7] as the GABAa-mediated excitation hypothesis. This transient change was performed with the synaptic weight of two of the three interneurons altered according to the following equation:

(21) φ−t=φ×0.25(tanh(t−ti0.2)+1.0)×(tanh(t−tf1.5)+1.0),

where *t*_*i*_ = 2 days indicates the onset of the inflammatory state and *t*_*f*_ = 12.5 days the end. Only two interneurons were submitted to the transient changes, since in the DG not all inhibitory synapses are GABAa [31]. The weight of the two interneuron connections was altered for the connection to 22 granule cells. This group of cells corresponds to the 22% of excitatory GABAa observed in epileptic human brain tissue [32, 33]. Similar findings were also encountered by Pathak et al. [30] studying DG granule cells in rat hippocampus.

**Figure 3:**
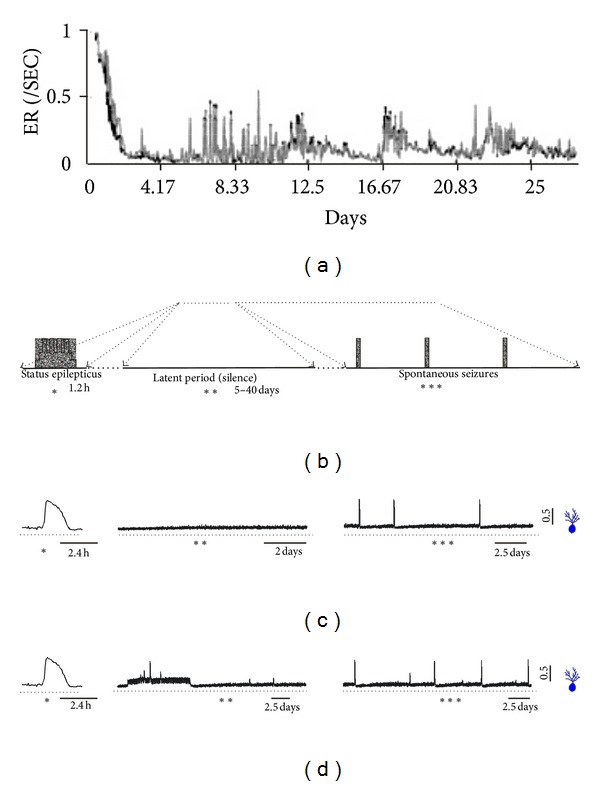
Comparison between simulations and experimental findings, showing the development of epileptogenesis and emergence of recurrent spontaneous seizures in models of *status epilepticus.* The *status epilepticus*, latent (or silent) period, and spontaneous seizures are depicted. (a) Event rate following kainic acid application (modified from van Puttena et al. [6]). (b) Diagrammatic representation of the periods (modified from Morimoto et al. [7]). (c) Average activity of the granule cells simulated with stimulation for inducing the *status epilepticus*. (d) Average activity of the granule cells simulating the *status epilepticus* and including change in the interneurons action from inhibitory to excitatory.

 By comparing the simulation with experimental data the model can be evaluated according to its ability to represent mechanisms subserving the synaptogenesis and epileptogenesis involved in the status epilepticus (Figure 3). The three main periods that characterize the seizure progression after status epilepticus can be distinguished in the simulations: status epilepticus, latent period, and spontaneous seizures. According to Morimoto et al. [7], in the model of status epilepticus the administration of chemical agents such as pilocarpine or kainate is followed by an emergence of continual recurrent seizures sustained for several hours. The same process is represented in the simulations with the network stimulation. Following the induction of status epilepticus, the absence of seizures characterizes the latent (or silent) period. When the seizure returns, the period of spontaneous seizures starts. The simulations involving the stimulation period are in close correspondence with the occurrence of these periods. In Figure 3, the event rates registered experimentally by van Puttena et al. [6] and a diagrammatic representation of the periods, according to Morimoto et al. [7], are correlated with the simulations.

**Figure 4:**
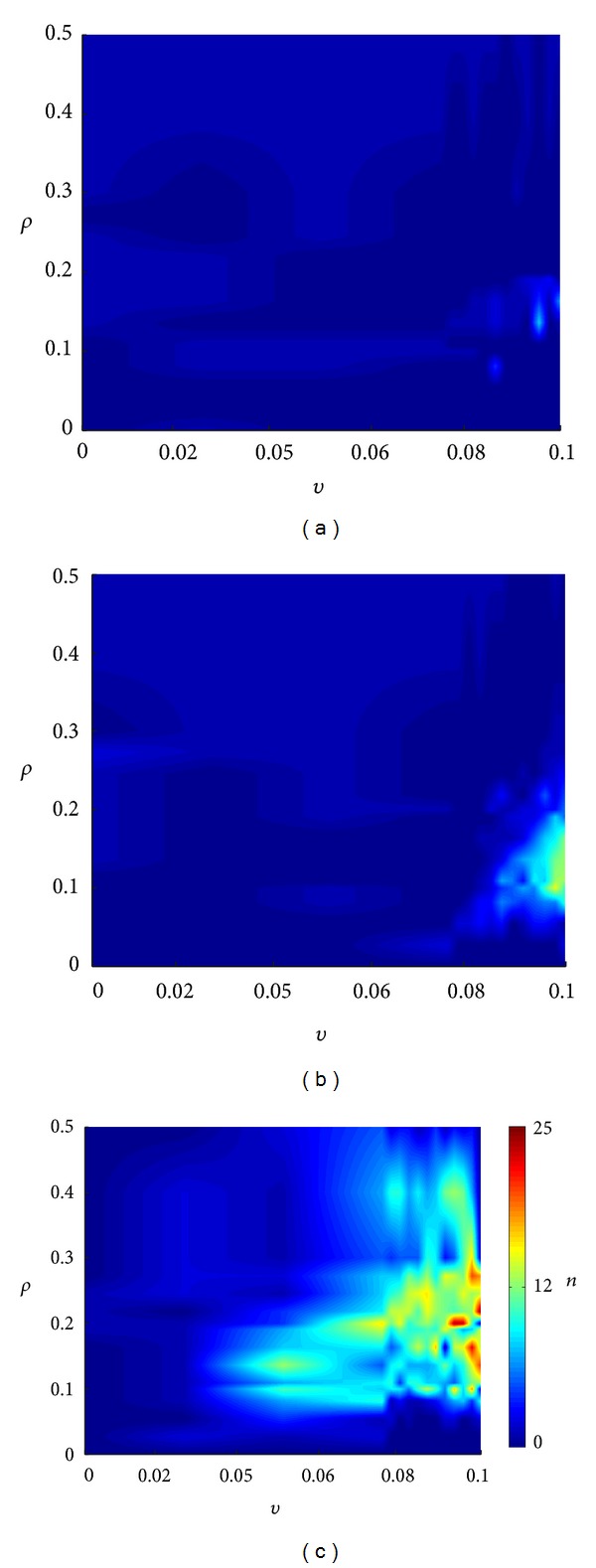
Spontaneous increases of SICA simulated for different values for *ρ* (Hebbian rules) and *v* (compensation theory). (a) No stimulation; (b) with stimulation; (c) with stimulation and the transient excitatory GABAa. “*n*” indicates numbers of SICA.

To analyze the SICA induction in the neuronal network, the occurrence of SICA along the 100 days, for all simulated situations, is shown in Figure 4. The SICAs are shown to have dependence on the combination of the parameters related to the compensation theory and the Hebbian rule. To perform the stimulation (Figure 4(b)), the stimulus was also applied at day 1.5, with *α* = 1.0, during 70 min. The demarked SICAs are the ones whose activity exhibited variations greater than 30%, assuming that the less intense activities are not necessarily related to epileptiform activities. The average activity of the cells and the excitation and inhibition functions showed similar behaviour during the SICA to those observed during the induced increase by stimulation (Figure 2). What distinguishes the two events is the manner of triggering the activity. The SICA deflagration was due to the increased efficacy of the synaptic connections.

In Figure 4(a), when the network is stimulated, it can be observed that the combinations of the compensation theory with Hebbian rule result in SICA for a limited range of values for *ρ* and *v*. In the case where the network was stimulated (Figure 4(b)), it is evident the increase of region where the SICA emerges, meaning larger range for *ρ* and *υ*. Furthermore, the number of SICA observed for each combination of *ρ* and *v* was higher. When the GABAa excitation was represented (Figure 4(c)), the conditions favorable for SICA occurred over a wider range of values of *ρ* and *v*.

**Figure 5:**
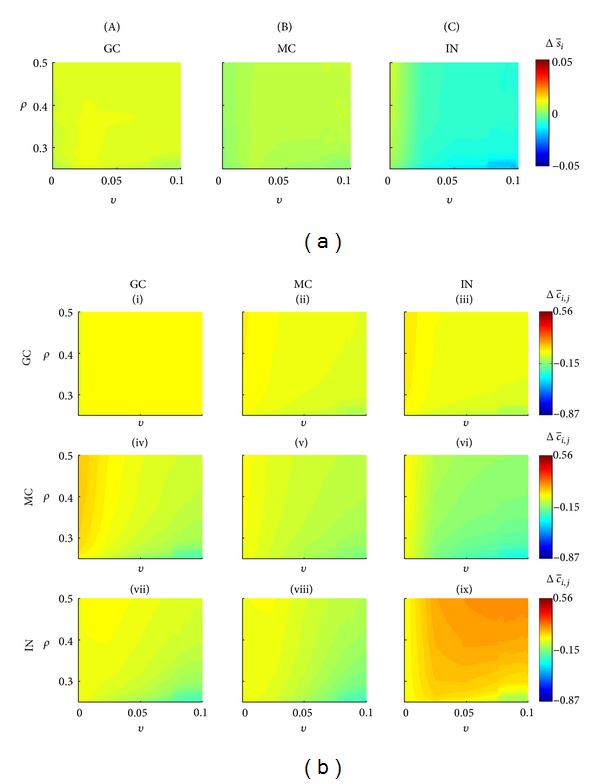
Network changes when no stimulation was applied, for different combinations of *ρ* (Hebbian rules) and *v* (compensation theory). (a) Percentage variation of the average activity for the three different cell types. (b) Connectivity changes for each group (I–IX). The changes in the average activity and connectivity were calculated for the first 41 days. GC: granule cells; MC: mossy cells; IN: interneurons.

In the situation where the network has not been stimulated, it is possible to investigate the average activity of the different cell types (Figure 5(a)) as well as for each group of connections (Figure 5(b)). After 41 days, applying the theory of compensation and the Hebbian rule caused variation in the average activity of granule cells that ranged between 0.0034 and 0.0096% (<1%). This variation is associated with small changes in the connectivity between the granular cells (group I), $\Delta {\overset{-}{c}}_{ij}\approx 0$. For most combinations of *ρ* and *v* (Figure 5(b), yellow region), there are changes in the connections that the mossy cells and the interneurons send to the granule cells (groups IV and VII, resp.). The major variations of the connectivity of the groups IV and VII occurred for higher values of *ρ* and lower values of *v* (Figure 5(b), light blue region). Reductions were observed for both groups of connection in the order of $\Delta {\overset{-}{c}}_{ij}\approx -0.25$. The changes in the average activity of the mossy cells were in the range −0.005 to 0.0068%, being associated with the following changes in the connectivity: group (II), $\Delta {\overset{-}{c}}_{ij}\in (-0.0981,0.0280)$; group (V), $\Delta {\overset{-}{c}}_{ij}\in (-0.1798,0.0150)$; and group (VIII), $\Delta {\overset{-}{c}}_{ij}\in (-0.2760,0.0060)$. Similarly for groups (I) and (IV), the major reductions of connectivity occurred for higher values of *v* and lower for *ρ* (Figure 5(b), light blue region). The change in the connectivity of the interneurons was in the range −0.0196 to 0.0079, and the reduction of the interneuron activity (Figure 5(a), IN, light blue region) can be attributed to the increased role of the compensation theory. The changes in the average activity were associated with the following connectivity changes: group (III), $\Delta {\overset{-}{c}}_{ij}\in (-0.1051,0.0393)$; group (VI), $\Delta {\overset{-}{c}}_{ij}\in (-0.3205,0.0330)$; and group (IX), $\Delta {\overset{-}{c}}_{ij}\in (-0.01341,0.1819)$.

**Figure 6:**
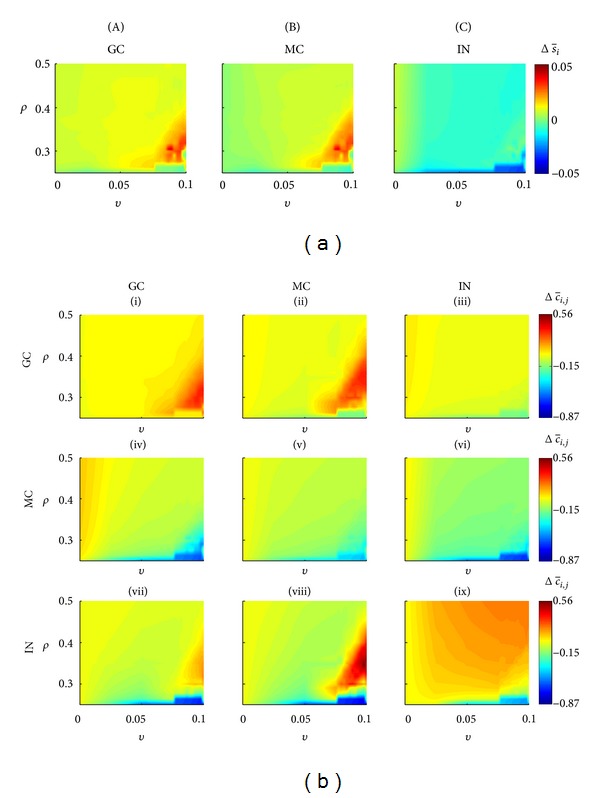
Network changes when stimulation was applied, for different combinations of *ρ* (Hebbian rules) and *v* (compensation theory). (a) Percentage variation of the average activity for the three different cell types. (b) Connectivity changes for each group (I–IX). The changes in the average activity and connectivity were calculated for the first 41 days. GC: granule cells; MC: mossy cells; IN: interneurons.

When the simulation includes the stimulation (*α* = 1.0, during 70 min), the percentage variation of the average activity in the 41st day, calculated in respect to the 1st day, was more intense for higher *v* values and lower *ρ* values (Figure 6(a), dark red regions, for GC and MC, and dark blue for IN). The increased average activity of the GC, in the order of 3% causing the most pronounced number of SICA for *ρ* ≤ 0.4 and *v* > 0.025, is associated with the connectivity increase of the groups (I) and (VII), respectively, $\Delta {\overset{-}{c}}_{ij}\approx 0.35$ and $\Delta {\overset{-}{c}}_{ij}\approx 0.19$, as well as reduction of the group (IV), with $\Delta {\overset{-}{c}}_{ij}\approx -0.60$ (Figure 6(b)). For the same combination of *ρ* and *v* values, the average activity of the MC also increased almost 3% and the following connectivity changes were observed: group (II), $\Delta {\overset{-}{c}}_{ij}\approx 0.34$; group (V), $\Delta {\overset{-}{c}}_{ij}\approx -0.44$; and group (VIII), $\Delta {\overset{-}{c}}_{ij}\approx 0.55$. On the other hand, the average activity of the interneurons decreased 3% and with the following connectivity changes with interneurons as the postsynaptic cells: group (III), $\Delta {\overset{-}{c}}_{ij}\approx -0.14$; group (VI), $\Delta {\overset{-}{c}}_{ij}\approx -0.16$; group (IX), $\Delta {\overset{-}{c}}_{ij}\approx 0.19$. When *ρ* = 0, that is, considering only the compensation theory, stimulation caused, especially for larger values of *v*, reduction in the level of activity and connectivity of the neurons.

**Figure 7:**
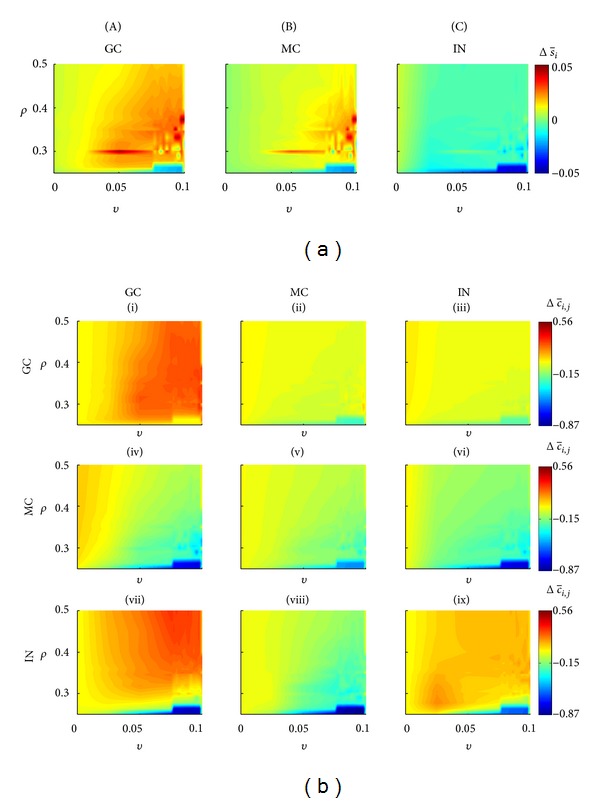
Network changes when stimulation was applied and the transient excitatory GABAa was represented, for different combinations of *ρ* (Hebbian rules) and *v* (compensation theory). (a) Percentage variation of the average activity for the three different cell types. (b) Connectivity changes for each group (I–IX). Changes in the average activity and connectivity were calculated for the first 41days. GC: granule cells; MC: mossy cells; IN: interneurons.

Including GABAa changing from inhibitory to excitatory for two interneurons and stimulating the neural network, the granule and mossy cells activities increase more intensely for a larger number of combinations of *ρ* and *v* values: *ρ* > 0.1 and *v* ≥ 0.0025 (Figure 7(a)). The increase of the average granule cell activities (~5%) increased the SICA occurrence for *ρ* > 0.1 and *v* ≥ 0.0025 and is associated with connectivity increase for the groups (I) $\left(\Delta {\overset{-}{c}}_{ij}\approx 0.32\right)$ and (VII) $\left(\Delta {\overset{-}{c}}_{ij}\approx 0.31\right)$ and connectivity decrease for the group (IV) $\left(\Delta {\overset{-}{c}}_{ij}\approx -0.74\right)$ (Figure 7(b)). For the same *ρ* and *v* values, the average activity of the mossy cells increased 4.5% and the following connectivity changes occurred: group (II), $\Delta {\overset{-}{c}}_{ij}\approx -0.15$; group (V), $\Delta {\overset{-}{c}}_{ij}\approx -0.2$; group (VIII), $\Delta {\overset{-}{c}}_{ij}\approx -0.50$. The average activity of the interneurons reduced ~4% and the following changes in the connectivity occurred for postsynaptic interneurons: group (III), $\Delta {\overset{-}{c}}_{ij}\approx -0.2$; group (VI), $\Delta {\overset{-}{c}}_{ij}\approx -0.25$; group (IX), $\Delta {\overset{-}{c}}_{ij}\approx 0.19$. When *ρ* = 0, therefore considering only the compensation theory, the stimulation followed by the transient excitatory GABAa caused the most intense activity and connectivity reduction (dark blue regions).

**Figure 8:**
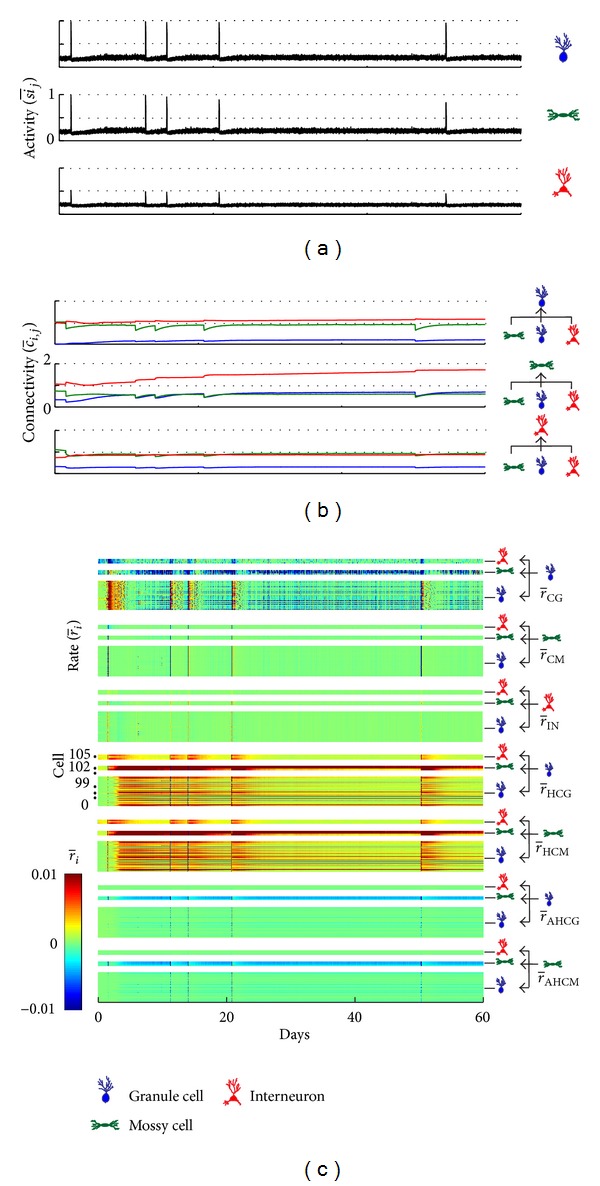
Average activity (a), average connectivity (b), and average synaptic formation rate for each cell type (c), depending on the Hebbian and anti-Hebbian rules and compensation theory. The simulation was performed with simulation at day 1.5, *v* = 0.1 and *ρ* = 0.2182.

The higher incidence of SICA, reproducing the induction of epileptiform activity in experimental models, occurred for *ρ* = 0.2182 and *v* = 0.1. For these combinations of the compensation theory with the Hebbian rule, the occurrence of SICA is depicted in Figure 8(a). Concomitantly, the inhibitory synapses formation rate increases throughout the network (Figure 8(c)—${\overset{-}{r}}_{\text{IN}}$). On the other hand, the connectivity of the excitatory synapses decreases in response to the activity increase (Figure 8(b)) due to the compensatory theory (Figure 8(c)—${\overset{-}{r}}_{\text{GC}},{\overset{-}{r}}_{\text{MC}}$) and the anti-Hebbian rule (Figure 8(c)—${\overset{-}{r}}_{\text{AH}}$). Following the afterdischarges induced by the network stimulation, between the second and twelfth days, the average activity of the network reduces (Figure 8(a)); it is when a progressive increase of the excitatory synapses takes place (Figure 8(b)—green and blue curves). These changes were correlated with the synaptic changes, with progressive increase of the excitatory synapses (Figure 8(b)), promoted by the Hebbian rule (Figure 8(c)—${\overset{-}{r}}_{H}$) and the compensation theory (Figure 8(c)—${\overset{-}{r}}_{\text{GC}},{\overset{-}{r}}_{\text{MC}}$), and decrease of the inhibitory synapses (Figure 8(b)), due to compensation. Later, synaptic activity increases until the emergence of SICA. Changes in activity and connectivity during and subsequent to the SICA were similar to those observed for the increased activity induced by stimulation. Furthermore, it can be seen that the connectivity of all groups of connections, including group (I) representing the interconnections between the granule cells, increases to a maximum level. 

**Figure 9:**
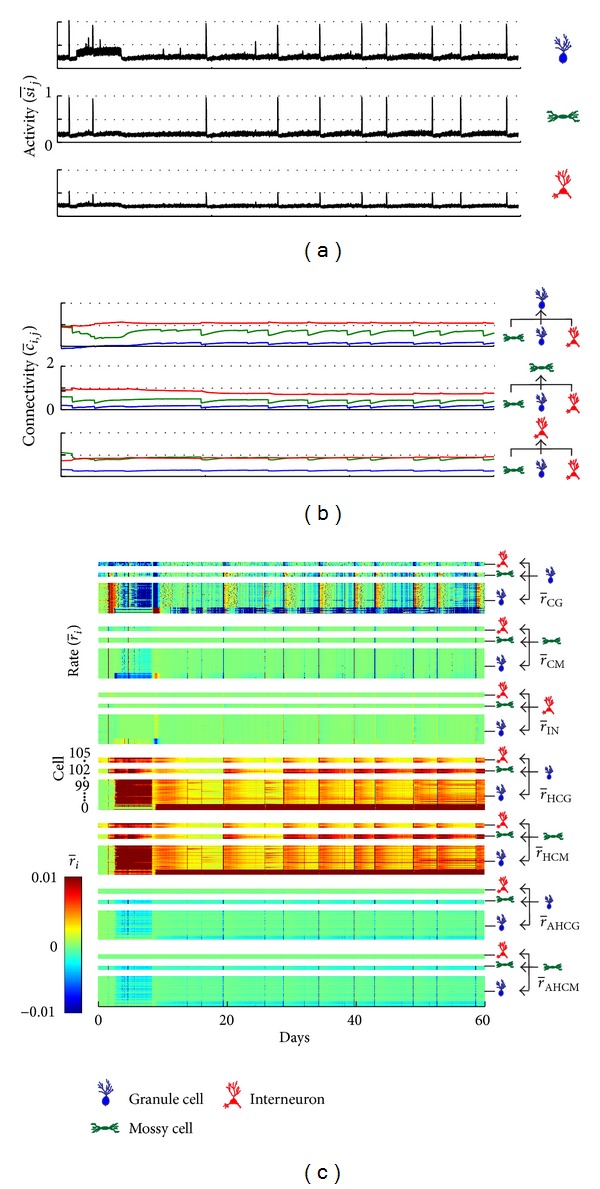
Average activity (a), average connectivity (b), and average synaptic formation rate for each cell type (c), depending on the Hebbian and anti-Hebbian rules and compensation theory. The simulation was performed with simulation at day 1.5, *v* = 0.1 and *ρ* = 0.2182, considering the occurrence of the transient excitatory GABAa.

When the simulation includes the transient excitatory GABAa, an activity increase (Figure 9(a)) after the activity induced by stimulation can be observed. During this period, it can be observed that (Figures 9(b) and 9(c)) (i) the connectivity between the granule cells increased simultaneously with the increase of the Hebbian rule rate (${\overset{-}{r}}_{H}$); (ii) connections sent by granule cells to other cell types were less intense; (iii) connectivity of the MC to the other cell types reduced due to the compensation theory (${\overset{-}{r}}_{\text{IN}}$); (iv) inhibitory connectivity increased for all cell types, due to compensation. After this period, a progressive increase of the connectivity takes place between the GC and also the excitatory connectivity of the MC. In respect to the inhibitory synapses, the main effect is reduction of the inhibitory input on the MC (Figure 9(b)). 

## 4. Discussion

The aim of the work presented was to investigate, by means of mathematical modelling and computational simulation, the interplay between changes in synaptic connectivity and the induction of SICA on neuronal networks that represents the circuitry of the DG of the rat hippocampus. The neuronal networks used described the cellular activity with dependence on the synaptic connectivity, according to McCulloch-Pitts formalism [15], and on the non-synaptic mechanisms typical of the DG region. The correspondence between the simulations and the experimental findings allows for the proposal of possible mechanisms involved. Assuming that the synaptic connectivity can be altered following the compensation theory [17, 18] and the Hebbian rules [24], the simulations performed suggest the following:

- the combined action of these synaptogenesis rules contributes to the occurrence of abnormal mossy fiber sprouting and SICA sustaining;
- besides the abnormal mossy fiber sprouting, other modifications of connectivity, such as those that result in reduction of inhibitory cell activity, contribute to the occurrence of SICA;
- the transient excitatory GABAa, during the inflammatory period subsequent to the *status epilepticus*, acts, intensifying the Hebbian rules action and, consequently, strengthening the abnormal mossy fiber sprouting.

### 4.1. Network in Absence of Stimulation

According to Schaefers et al. [34], the DG exhibits high synaptic turnover rate and has a highly dynamic structure not restricted to the prenatal ontogenesis. These properties are also maintained after birth, during development, and can also take place in an adult brain. Mimicking these features in the simulations, the fluctuation of the activities induces the synaptogenesis processes. Without stimulation, the network remains in equilibrium for almost all combinations of the compensatory theory with the Hebbian rules. Only in a narrow range of lower *ρ* values and higher *v* do the SICA increase (Figure 4). These simulations suggest that if alterations on the brain tissue reinforce the role of compensation theory and depress the Hebbian rules' action, the network will evolve in time favouring the emergence of epileptiform events, even in the absence of stimulation or another action to increase excitation. In this situation, two alterations in the connectivity can be qualified as the main reason for the SICA (Figure 5): (i) the decrease of the inhibition action on the mossy cells and (ii) the increase of the inhibition action on the interneurons. 

The first alteration in the connectivity contributes to the emergence of the SICA, which in turn enhances the activation of the granule cells. This mechanism indicated by the simulations is consistent with the hypothesis formulated by Santhakumar et al. [35]. This hypothesis, named “irritable mossy cells hypothesis,” proposes that the increase of the mossy cell excitability is responsible for the generation and sustaining of epileptic seizures. 

The increase of the inhibitory action on the interneurons, therefore the second connectivity alteration, results in granule and mossy cells disinhibition. This action is also favourable to the SICA emergence. This mechanism can be associated with the recurrent inhibition hypothesis, suggested by Sharma et al. [3], and also to explain the generation and sustaining of the epileptic seizures. In this hypothesis, the interneurons' death or activation reduction is the main cause of the seizure disruption. The simulations present additional mechanisms that can produce the same effect. The increase of the interconnectivity between interneurons, aiming at compensating the network activity changes, can also contribute to reduce the inhibition efficacy, enhancing the granule cell excitation and therefore the seizures. 

In the simulations, without stimulation, the SICAs were not due to the abnormal mossy fibers sprouting, since there was no significant increase in the interconnectivity of the granule cells (Figure 4—group (I)). This indicates that the abnormal mossy fiber sprouting, although often observed in experimental epilepsy [36], is not necessary for the seizure disruption. This result is consistent with the experimental findings from Buckmaster and Lew [37]. The epileptic rats treated with rapamycin, a suppressant of the axon sprouting, had no effect on seizure occurrence. It has been shown that amygdala, subiculum, and other temporal limbic structures could replace the mossy fiber effects. In fact, other brain areas show increases in recurrent excitation in models of TLE. The simulations offer an additional insight into this field, the network disconnected from other structures, and without mossy fiber sprouting was able to support synaptic reorganization with generation of spontaneous seizure by means of the Hebbian rules and compensation theory.

### 4.2. Network with Stimulation

When stimulated, the network simulation shows an increase in the neuronal activity enough to overcome the threshold for actuation of nonsynaptic mechanisms with subsequent triggering of SICA (Figure 2). The SICA duration was about 1.8 h and, associated with the experimental conditions, represents the *status epilepticus*. According to the simulations, the *status epilepticus* may be modulated by nonsynaptic mechanisms. In fact, experimental observations offer support to this speculation, since it was confirmed that nonsynaptic mechanisms are able to sustain epileptiform seizures in DG of rats' hippocampus [14, 22, 38–40].

During the period corresponding to the *status epilepticus*, the compensation theory causes excitatory reduction and inhibitory increase in the network connectivity. In consequence, immediately after the end of the period, the activity of the neural network was reduced, characterized as the latency period for the occurrence of SICA, which may be associated with the latency period observed for epilepsy induction with experimental models [7, 41]. During this period, following the compensation theory, new excitatory synapses are formed with the goal of reversing the activity reduction. Among these excitatory synapses included the interconnectivity between granule cells and the increase which characterizes the abnormal mossy fiber sprouting. The simulations suggest that the abnormal mossy fiber sprouting starts as a protective mechanism of the neural network, in order to compensate the increase of inhibitory synapses that occur during *status epilepticus*. This hypothesis reinforces the view that the axonal sprouting and the reactive synaptogenesis of the dentate gyrus serve as a repair mechanism, in order to restore normal function of the tissue [36]. However, an increase in the interconnectivity of the granule cells improves the performance of the Hebbian rules and the firing of a pre-synaptic cell is able to influence even more the firing of the postsynaptic cell connected. Consequently, the simulations suggest that the Hebbian rules, reinforced by the compensation theory, contribute to intensify the abnormal mossy fiber sprouting. This may happen along several days, during the latency period and even after SICA disruption. The intense interconnectivity between granule cells (Figures 5(b) and 7(b)) and the resulting increase of SICA occurrence (Figure 4(b)) take place for higher *v* values (compensation theory) and lower, however not null, *ρ* value (Hebbian rule), supporting the hypothesis that both theories (compensation and Hebbian) may be realized in the same network [19]. This dependence on *ρ* and *v* suggests that the interconnectivity increases between granule cells (abnormal mossy fiber sprouting) and the SICA happens only if the compensation theory forms inhibitory synapses and degenerates excitatory synapses more efficiently than the Hebbian rules increaseing the synaptic weight of the excitatory connections of the network. Additionally, the simulations show that the Hebbian rules are indispensable. When *ρ* = 0, the stimulation leads the network to a degeneration due to the action of the compensation theory. 

### 4.3. Network with Stimulation and Transient Excitatory GABAa

The simulation of the network including the stimulation and also the excitatory effect of the GABAa synapses increased the occurrence of SICA and the range of combining values for *ρ* and *v* able to sustain them. The main changes observed in these simulations were the intense increase of the interconnectivity between granule cells (Figures 7 and 9). The simulations suggest that the excitatory GABAa synapses counteract the activity reduction caused by the increase of inhibitory connectivity along of the *status epilepticus*. This process enhanced granule cell firing, which, after the *status epilepticus*, was more interconnected due to the compensation theory. Thus, the granule cell promotes even more firing of action potentials between them. Therefore, the Hebbin rules were reinforced and the abnormal mossy fiber sprouting was intensified. These simulations support the conjecture that slow plasticity changes (Hebbian plasticity) after brain injury or *status epilepticus* may act on the eventual transition to the epilepsy state [42].

## 5. Conclusion

**Figure 10:**
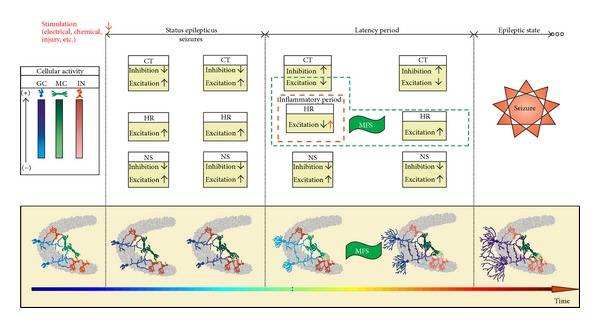
Pictorial representation of the conjoint action of the compensation theory and the Hebbian rules for the structural rearrangement of the dentate gyrus favourable to epileptic seizures. (bottom row) DG circuitry represented schematically with connectivity level (axonal branches) and cell activity (colour intensity). (top row) Excitation and inhibition actions associated to the compensation theory (CT), the Hebbian rules (HR), and the nonsynaptic mechanisms (NS). The neuronal network states associated with the epilepsy induction states (resting, *status epilepticus*, latent, and epilepsy) are indicated in the top line. The inflammatory period is indicated and its effect on the Hebbian rules is highlighted in red. The mechanisms involved in the abnormal mossy fiber sprouting are indicated by the green dashed line. GC: granule cells; MC: mossy cells; IN: interneurons.

The simulations of the present paper allow speculation about the conjoint action of the compensation theory and the Hebbian rules during *status epilepticus* and the latency period promoting synaptic circuitry changes favourable to spontaneous seizure (Figure 10). According to the simulations, the network stimulation (electrical, chemical, injury, etc.) increases the neuronal activity leading to seizure mimicking the *status epilepticus.* During *status epilepticus*, seizures are strongly modulated by nonsynaptic mechanisms that sustain the depolarization even with the synaptic circuitry changes promoted by the compensation theory. The Hebbian rules take place, strengthening the current excitatory connections. When the nonsynaptic mechanisms of inhibition, in the case the Na/K pump, act to overcome the excitation mechanisms (e.g., current Na^+^ channels), the *status epilepticus* ends. From that moment, the excitability shows a dramatic reduction characterizing the beginning of the latency period. At the beginning of this period the compensation theory acts, reducing inhibition and increasing excitation. Contributing particularly to the excitation increase the simulations show the emergence of excitatory connections between granule cells, which indicates the onset of abnormal mossy fiber sprouting. During this period, the non-synaptic mechanisms do not contribute significantly to the excitability. In this sense, the abnormal mossy fiber sprouting arises as a protective mechanism, which tends to reverse the reduction of granule cell activities induced by the *status epilepticus*. The mossy fiber sprouting increases the connectivity between granule cells and reinforces the action of the Hebbian rules. In positive feedback, the Hebbian rule enhances the mossy fiber sprouting and helps to increase granule cell activity. Additionally, at the beginning of the latency period, the effect of the transient excitatory GABAa, promoted by the inflammatory process, contributes to reverse the neuronal activity reduction. This effect further enhances the performance of the Hebbian rules on the mossy fiber sprouting. As long as the abnormal mossy fiber sprouting intensifies, the excitation increases and, following the compensation theory, the network reacts to reverse this situation. When the abnormal mossy fiber sprouting is able to increase the granule cell activities overcoming the threshold of the nonsynaptic mechanisms actuation, the transition to the epilepsy state takes place. 

The predictions proposed in this paper could be used to experimentally test the conjoint action of the compensation theory and Hebbian rule on the epileptogenesis induced by models of *status epilepticus.*

## Figures and Tables

**Table 1:** Morphogenetic rules according to Dammasch et al. [21].

Neurons in high state of activity, *H*, when *s*_*i*_^*t*^ > 0.25	Neurons in low state of activity, *L*, when *s*_*i*_^*t*^ < 0.15
Δσ_*j*_^bpr^ = 0	Δσ_*j*_^bpr^ = −*k*_bpr_^*L*^|Δ*s*_*i*_^*t*^| < 0
Δσ_*i*_^bepo^ = −*k*_bepo_^*H*^|Δ*s*_*i*_^*t*^| < 0	Δσ_*i*_^bepo^ = 0
Δσ_*i*_^bipo^ = 0	Δσ_*i*_^bipo^ = −*k*_bipo_^*L*^|Δ*s*_*i*_^*t*^| < 0
Δσ_*j*_^fpr^ = *k*_fpr_^*H*^|Δ*s*_*i*_^*t*^| > 0	Δσ_*j*_^fpr^ = −*k*_fpr_^*L*^σ_*i*_^fpr^|Δ*s*_*i*_^*t*^| ≤ 0
Δσ_*i*_^fepo^ = −*k*_fepo_^*H*^σ_*i*_^fepo^|Δ*s*_*i*_^*t*^| ≤ 0	Δσ_*i*_^fepo^ = *k*_fepo_^*L*^|Δ*s*_*i*_^*t*^| > 0
Δσ_*i*_^fipo^ = *k*_fipo_^*H*^|Δ*s*_*i*_^*t*^| > 0	Δσ_*i*_^fipo^ = −*k*_fipo_^*L*^σ_*i*_^fipo^|Δ*s*_*i*_^*t*^| ≤ 0
